# 2406. A Changing Landscape: Trends in Infective Endocarditis in Western India - A Single Centre Retrospective Analysis

**DOI:** 10.1093/ofid/ofad500.2026

**Published:** 2023-11-27

**Authors:** Santhanam Naguthevar, Akshatha Ravindra, Gopal Krishana Bohra, Deepak Kumar, Durga Shankar Meena, Vibhor Tak, Subhashree Samantaray, Shivang Sharma, M K Garg

**Affiliations:** AIIMS JODHPUR, Jodhpur, Rajasthan, India; AIIMS Jodhpur, Jodhpur, Rajasthan, India; AIIMS Jodhpur, Jodhpur, Rajasthan, India; All India Institute of Medical Sciences, Jodhpur (India), Jodhpur, Rajasthan, India; AIIMS, Jodhpur, Rajasthan, India; AIIMS, Jodhpur, Rajasthan, India; AIIMS, Jodhpur, Rajasthan, India; AIIMS, Jodhpur, Rajasthan, India; AIIMS, Jodhpur, Rajasthan, India

## Abstract

**Background:**

Infective endocarditis (IE) is a life-threatening infection that poses a significant risk to morbidity and mortality. Traditionally, IE has been associated with pre-existing cardiac diseases such as congenital heart disease (CHD) or rheumatic heart disease (RHD). This study aims to describe the changing epidemiology of IE in patients admitted to a tertiary care centre in Western India.

**Methods:**

We conducted a prospective view of cases of definite IE using the Modified Duke Criteria. Our study analysed patient records from April 2021 to March 2023, and we collected demographic data, clinical manifestations, laboratory findings, microbiological data, treatment modalities, and outcomes.

**Results:**

A total of 50 cases of definite IE were identified according to the modified Duke’s criteria. The patients had a mean age of 36±17 years, with males being more affected (75%). Prior heart diseases were identified as a predisposing factor in 50% of cases, with rheumatic heart disease (60%) and congenital heart disease (40%) being the most common. Notably, two patients had IE secondary to coronary stent infection.

Among the 74% culture-positive cases, Staphylococcus aureus (24.3%) was the most frequently isolated organism, with methicillin-resistant S. aureus (44.4%) and Pseudomonas aeruginosa (13.5%) being the predominant pathogens. Other organisms isolated included Enterococcus spp. (n=4), MSCONS (n=4), Candida albicans (n=3), Mycobacterium abscessus (n=1), and Brucella melitensis (n=1).

In the culture-negative endocarditis group (26%), 15.4% were diagnosed with non-bacterial thrombotic endocarditis (NBTE). The mitral valve (66%) was the most commonly affected valve, with the majority (58%) showing large vegetation (≥10mm). Complications such as central nervous system embolization were frequently observed (40%), and mortality occurred in 20% of cases

**Conclusion:**

Although pre-existing heart disease, including rheumatic heart disease and congenital heart disease, remains the most common risk factor for IE, there has been a noticeable trend of increasing IE in prosthetic devices. In recent years also Staphylococcus aureus only the leading organism for IE.

**Disclosures:**

**All Authors**: No reported disclosures

